# Kartogenin Enhances Chondrogenic Differentiation of MSCs in 3D Tri-Copolymer Scaffolds and the Self-Designed Bioreactor System

**DOI:** 10.3390/biom11010115

**Published:** 2021-01-16

**Authors:** Ching-Yun Chen, Chunching Li, Cherng-Jyh Ke, Jui-Sheng Sun, Feng-Huei Lin

**Affiliations:** 1 Department of Biomedical Sciences and Engineering, National Central University, Taoyuan 32001, Taiwan; chingyun523@gmail.com or; 2 Institute of Biomedical Engineering, College of Medicine and College of Engineering, National Taiwan University, Taipei 10002, Taiwan; R01548007@ntu.edu.tw; 3 Biomaterials Translational Research Center, China Medical University Hospital, Taichung 40202, Taiwan; fonchanwd@gmail.com; 4 Center for General Education, China Medical University, Taichung 40202, Taiwan; 5 Master Program for Digital Health Innovation, China Medical University, Taichung 40202, Taiwan; 6 Master Program in Technology Management, China Medical University, Taichung 40202, Taiwan; 7 Department of Orthopedic Surgery, National Taiwan University Hospital, Taipei 10002, Taiwan; 8 School of Medicine, College of Medicine, China Medical University, Taichung 40202, Taiwan; 9 Institute of Biomedical Engineering and Nanomedicine (I-BEN), National Health Research Institutes, Miaoli 35053, Taiwan

**Keywords:** kartogenin, chondrogenic differentiation, MSCs, 3D culture, tri-copolymer scaffolds, self-designed bioreactor system

## Abstract

Human cartilage has relatively slow metabolism compared to other normal tissues. Cartilage damage is of great clinical consequence since cartilage has limited intrinsic healing potential. Cartilage tissue engineering is a rapidly emerging field that holds great promise for tissue function repair and artificial/engineered tissue substitutes. However, current clinical therapies for cartilage repair are less than satisfactory and rarely recover full function or return the diseased tissue to its native healthy state. Kartogenin (KGN), a small molecule, can promote chondrocyte differentiation both in vitro and in vivo. The purpose of this research is to optimize the chondrogenic process in mesenchymal stem cell (MSC)-based chondrogenic constructs with KGN for potential use in cartilage tissue engineering. In this study, we demonstrate that KGN treatment can promote MSC condensation and cell cluster formation within a tri-copolymer scaffold. Expression of *Acan*, *Sox9*, and *Col2a1* was significantly up-regulated in three-dimensional (3D) culture conditions. The lacuna-like structure showed active deposition of type II collagen and aggrecan deposition. We expect these results will open new avenues for the use of small molecules in chondrogenic differentiation protocols in combination with scaffolds, which may yield better strategies for cartilage tissue engineering.

## 1. Introduction

As an aneural, avascular, alymphatic, and hypocellular tissue, articular (hyaline) cartilage has been the primary focus of most clinicians. The dry weight of articular cartilage is mainly composed of type II collagen and proteoglycans, which provide load-bearing function [1]. Chondrogenic tissues in normal conditions have a relatively slow potential of healing; damage to cartilage is of great clinical concern since the cartilage tissue has limited intrinsic healing potential. Cartilage lesions arising due to aging, joint laxity, excessive stress imposed upon normal tissue, diet, hormones, crystal deposition, bone microfractures, and immunologic factors have been implicated in the etiopathogenesis of osteoarthritis [2]. Osteoarthritis (OA) is not the result of diminished metabolic activity; on the contrary, it is a very active catabolic process [3]. Synthesis of cartilage matrix and cell division occurs at greater rates in damaged tissue than in normal cartilage tissue. However, the heightened synthesis activity irritates lysosomes, which results in the release of lysosomal enzymes that degrade cartilage and a reduction in proteoglycan contents in proportion to the disease severity. The matrix degradation rates of cartilage eventually exceed the rate of synthesis; thereby, the cartilage becomes eroded and forms lesions [1].

The mechanism of cellular synthesis and secretion of cartilage extracellular matrix (ECM) can be regulated and promoted by signaling from surrounding ECM molecules. In a previous study, we had synthesized a gelatin-chondroitin-6-sulfate-hyaluronan tri-copolymer to mimic natural cartilage matrix; ECM secretion with lacunae formation, and production of glycosaminoglycans (GAGs) makes tri-copolymer a promising scaffold for cartilage tissue engineering [4]. However, using chondrocytes as a cell source still caused morbidity at the donor site, making it impractical to use in the clinic. Current clinical therapies for cartilage repair are still less than satisfactory and rarely recover full function or return the pathological tissue to its native normal state [5,6].

Full-thickness cartilage lesions are found frequently, but the treatment of such articular defects has continued to be a challenge, with no traditional medical treatment providing the desired persistent, long-term efficacy [7,8]. In contrast, marrow stimulation techniques, such as drilling or microfracture, have been merely used to treat relatively small defect sites with the injection of multipotent stem cells, whereas the implantation of cultured autologous cells or engineered tissue constructs for full-thickness cartilage lesions serves a better option to hyaline-like cartilage regrowth. Cell-based regenerative therapy is a novel method of surgical treatments and is indicated for cartilage repair, and it plays a major role in the medical treatment of cartilage-associated diseases [9,10,11]. To date, these treatments have had limitations, and their outcomes are still inconsistent. Disadvantages such as the persistent morbidity on donor site, fibrous cartilage formation, lack of integration, and loss of cell viability due to graft storage have limited their clinical applications [12,13]. In short, the main challenges for cartilage reconstruction include selecting appropriate cell sources, choosing suitable scaffolds, creating biomechanically adequate tissues, and integrating to native tissue after implantation remain elusive [10,14].

MSCs form a specific cell population with highly regulated self-renewing ability; MSCs secrete a wide spectrum of bioactive molecules, including growth factors and cytokines, to avoid allogeneic rejection; thus, MSCs can be considered as an ideal cell source for therapeutic use that may open new frontiers in medicine [7,15]. The secreted bioactive factors offer a regenerative microenvironment at defect sites to restrict the area of damage and aid in regenerating tissues by communicating with resident cell populations at the defect site. The adult MSC is culture-dish adherent, and thus, it can be easily isolated from bone marrow aspirates and can be expanded in culture while preserving its multipotency. MSCs have been largely used in preclinical trials for tissue engineering, and they hold considerable promise for therapeutic use in repairing and in reconstructing damaged or diseased mesenchymal tissues [16,17].

A small molecule, kartogenin (KGN), can promote chondrocyte differentiation in both in vitro and in vivo animal models. Due to KGN is a nonprotein chondrogenesis inducing agent, it has excellent stability and can be transported at room temperature [18]. In other words, KGN is suitable for clinical use. Previous studies have demonstrated that KGN regulates the Runt-related transcription factor 1 (*CBFβ-Runx1*) transcriptional program to induce chondrogenesis [19,20,21,22]. It can stimulate MSCs to differentiate as matrix-producing chondrocytes while up-regulating type II collagen (*Col2a1*), SRY (sex determining region Y)-box 9 (*Sox9*), Aggrecan (*Acan*), and tissue inhibitor of metalloproteinase (*TIMPs*) expression; meanwhile, KGN also down-regulates Runt-related transcription factor 2-related (Runx2-related) downstream genes, thereby preventing chondrocyte hypertrophy [20,23]. However, the efficacy of KGN, along with a biomaterial scaffold, has not yet been assessed. Recently, some researchers tried to combining KGN with biomaterial scaffold for facilitating effective cartilage repair [18,22,24]; however, KGN worked in a dose-dependent manner for cartilage regeneration [25]. In the present study, we aim to optimize the chondrogenic process in MSC-based chondrogenic constructs. For this, we cultured rat MSCs on 3D tri-copolymer porous scaffolds with KGN in the dynamic self-designed bioreactor system and studied the effects on chondrogenic differentiation at the cellular and molecular levels.

## 2. Materials and Methods

### 2.1. Isolation of Rat MSCs

The methods were carried out in accordance with the approved guidelines (NTU- IACUC Approval No. 20140040) for animal experimentation by the Institutional Animal Care Committee, National Taiwan University College of Medicine. The MSCs of male Wistar rats were isolated using their plastic adherence [26]. Briefly, eight rats were sacrificed (eight weeks old) by carbon dioxide (CO_2_) asphyxiation, femurs and tibias were dissected aseptically and removed associated soft connective tissues extensively. The distal ends of femurs and tibias were opened, and the marrow cavities were flushed with phosphate buffered saline (PBS, pH 7.4). The mononuclear cell (MNC) fraction was isolated according to standard techniques by using a sterile density gradient media, Ficoll-Paque PLUS (an aqueous solution of density 1.077 ± 0.001 g/mL, GE Healthcare, Buckinghamshire, UK), and centrifuging around 300 xg at 20 °C for 40 min. The isolated cells were washed with PBS three times and resuspended in low glucose Dulbecco’s Modified Eagle’s medium (LG-DMEM, Life Technologies, Grand Island, NY, USA) supplemented with 10% fetal bovine serum (FBS, Biological Industries, Beit-Haemek, Israel). The cells were cultured at 37 °C in 5% CO_2_ atmosphere for three days. After 72 h incubation, the non-adherent cells were removed by washing with PBS gently and leave behind the adherent cell population to grow. When reaching 80% confluence, trypsinize was used, and cells were subcultured for expanding. In this study, the rat mesenchymal stem cells (rMSCs) were used at passages two to four throughout the following experiments.

### 2.2. Osteogenic, Chondrogenic, Adipogenic Differentiation Evaluation of rMSCs

To induce osteogenic differentiation, rMSCs (passage P2) were seeded at 5 × 10^3^ cells/cm^2^ on tissue culture plastic plates and cultured in an osteogenic medium. The osteogenic medium consisted of LG-DMEM supplemented with 2% FBS, 1% PSA, 0.1 μM dexamethasone (Dex, Sigma-Aldrich, St. Louis, MO, USA), 0.2 mM L-ascorbic acid 2-phosphate (Sigma-Aldrich, St. Louis, MO, USA), and 10 mM β-glycerophosphate (Sigma-Aldrich, St. Louis, MO, USA). These cells were cultured at 37 °C in 5% CO_2_ atmosphere for 14 days, and the medium was replaced every two days.

To induce chondrogenic differentiation, rMSCs (passages P2) were seeded at 5 × 10^5^ cells/drop on non-coating plastic plates to form a pelleted micro-mass and cultured in chondrogenic medium. The chondrogenic medium consisted of LG-DMEM supplemented with 2% FBS, 1% PSA, 0.1 μM dexamethasone, 50 μg/mL L-ascorbic acid 2-phosphate, 100 μg/mL sodium pyruvate (Sigma-Aldrich, St. Louis, MO, USA), 40 μg/mL proline (Sigma-Aldrich, St. Louis, MO, USA), 10 ng/mL Transforming Growth Factor Beta 2 (TGF-β2, Invitrogen), and 50 mg/mL ITS^+^ premix (6.25 μg/mL insulin, 6.25 μg/mL transferrin, 6.25 ng/mL selenius acid, 1.25 mg/mL bovine serum albumin, and 5.35 mg/mL linoleic acid; Sigma-Aldrich, St. Louis, MO, USA). These cells were cultured at 37 °C in 5% CO_2_ atmosphere for 14 days, and the medium was replaced every two days.

To induce adipogenic differentiation, rMSCs (passages P2) were seeded at 1 × 10^4^ cells/cm^2^ on tissue culture plastic plates and cultured in an adipogenic medium. The adipogenic medium consisted of LG-DMEM supplemented with 10% FBS, 1% PSA, 10 mg/mL insulin (Sigma-Aldrich, USA), 0.2 mM indomethacin (Sigma-Aldrich, USA), 1 mM Dex, 0.5 mM 3-isobutyl-1-methylxanthine (IBMX, Sigma-Aldrich, USA). These cells were cultured at 37 ℃ in 5% CO_2_ atmosphere for 14 days, and the medium was replaced every two days.

### 2.3. rMSC Characteristic Analysis by Flow Cytometry (FC)

The immunophenotypic analysis of rMSCs was carried out using direct staining protocols with conjugated monoclonal antibodies using a flow cytometry method. The isolated cells of passage three were characterized with respect to the expression of surface antigens. The expression of the following four surface antigens: CD45 (BD Biosciences, San Jose, CA, USA), CD29 (BD Biosciences, San Jose, CA, USA), CD44 (BD Biosciences, San Jose, CA, USA), and CD90 (BD Biosciences, San Jose, CA, USA) were characterized confirmed by LSR II flow cytometer with 488 nm laser option (BD Biosciences, San Jose, CA, USA). The data were analyzed with the FlowJo software (Treestar, Ashland, OR, USA). The forward and side scatter (FSC/SSC) profiles were utilized to distinguish between the signals of the cell population and to gate out debris or dead cells.

### 2.4. Synthesis of Tri-Copolymer Scaffolds

The fabrication of gelatin-chondroitin-hyaluronan tri-copolymer scaffolds was performed according to Chang et al. [4], following the percentage dry weight of each component of hyaline cartilage. Briefly, 0.5 gm gelatin powder (Sigma-Aldrich, St. Louis, MO, USA), 5 mg sodium hyaluronate (HA) powder (Sigma-Aldrich, St. Louis, MO, USA), and 0.1 gm chondroitin-6-sulfate (C6S) powder (Sigma-Aldrich, St. Louis, MO, USA) were mixed within 7.5 mL double-distilled water and cross-linked for 2–3 min at room temperature by using 1% 1-ethyl-3-(3-dimethylaminopropyl) carbodiimide (EDC, Sigma-Aldrich, St. Louis, MO, USA) at pH 5–6. The complex was injected into 48-well culture plates, frozen under −20 °C for 1 h, transferred to −80 °C for 1 h, and then lyophilized for 72 h by freeze-drying technique. The dried scaffold was re-cross linked for 48 h at room temperature by using 0.2% EDC, sterilized with 75% alcohol, then lyophilized for 72 h. A tri-copolymer scaffold about 5 mm in diameter and 5 mm in height was produced for the following experiments.

### 2.5. Measurement of Tri-Copolymer Scaffolds Cross-Linking Degree

In order to estimate the degree cross-linking in tri-copolymer scaffolds, the TNBS assay was applied by measuring the concentration of residual amine groups. The free amino group contents of tri-copolymer scaffolds were determined by ultraviolet-visible spectroscopy (UV/Vis, SpectraMax M5, Molecular Devices, Silicon Valley, CA, USA) after TNBS (Sigma-Aldrich, St. Louis, MO, USA) interaction [27,28]. Briefly, the non-cross linked and cross-linked tri-copolymer scaffolds were separately dissolved in 0.1 M sodium bicarbonate solution (pH 8.5) at a concentration of 200 μg/mL during different time periods. After incubation, 0.25 mL of the 0.01% (*v/v*) TNBS/sodium bicarbonate solution was added to 0.5 mL sample solution followed by reacting at 37 °C for 2 h. About 0.25 mL of 10% sodium dodecyl sulfate (SDS, Sigma-Aldrich, St. Louis, MO, USA) solution and 0.125 mL of 1 N hydrochloric acid (HCl, Sigma-Aldrich, St. Louis, MO, USA) solution were added to each sample, and the absorbance was measured at a wavelength of 345 nm. The measurement of degradation was performed by detecting the TNBS-labeled amino groups richly present in gelatin. In this study, L-lysine was used as the standard.

### 2.6. Self-Designed Bioreactor System

Bioreactor design and operation were described previously [29,30,31,32] and were carried out using the closed-loop bioreactor system. Briefly, the bioreactor system could separate into the cell culture tank and culture medium tank. The cell culture tank was composed of a 50 mL centrifuge tube (sterile) and a glass casing pipe (for mass transportation). The culture medium tank is composed of a 500 mL glass bottle and a GL45 plastic cap. The GL45 plastic cap was comprised of four stainless ports for culture medium transportation and gas perfusion. All consumables of this system were sterilizable via 121 °C autoclaving. The bioreactor system was located inside an incubator with high humidity in the atmosphere (37 °C, 5% CO_2_). The flow rate was set at 1 mL/min via a peristaltic pump (Longer, Baoding, China), which provided continuous culture medium replenishment.

### 2.7. Cell Seeding and Culture in the Self-Designed Bioreactor System

rMSCs were suspended in medium and then seeded into scaffolds at a density of 1 × 10^7^ cells/mL. The tri-copolymer scaffold/rMSCs constructs were placed in a culture plate for 24 h for cell adhesion, then either cultured in static condition for seven days or cultured in the self-designed bioreactor system up to 21 days. After 24 h for cell adhesion, all the constructs were cultured with chondrogenic medium. The chondrogenic medium contained LG-DMEM, 10% FBS, 1x ITS liquid media supplement (Sigma-Aldrich, St. Louis, MO, USA), 50 µg/mL ascorbic acid (Sigma-Aldrich, St. Louis, MO, USA), 40 µg/mL proline (Sigma-Aldrich, St. Louis, MO, USA), 100 µg/mL sodium pyruvate (Sigma-Aldrich, St. Louis, MO, USA), and 0.1 µM or 1.0 µM of KGN (Merck, Darmstadt, Germany). At each time period, 5 mL of the medium were sampled for GAGs quantification (*n* = 3) via dimethylmethylene blue (DMMB, Sigma-Aldrich, St. Louis, MO, USA) assay.

### 2.8. Scanning Electron Microscopy (SEM)

The scaffold and MSC morphology inside tri-copolymer scaffolds was observed by scanning electron microscopy (SEM) (TM 3000, Hitachi, Tokyo, Japan). Briefly, cells in scaffolds were fixed with 4% para-formaldehyde (PFA, Affymetrix, Santa Clara, CA, USA) for 2 h and 2% osmium tetroxide (OsO_4_, Sigma-Aldrich, St. Louis, MO, USA) solution for 1 h. All the samples were dehydrated in a graded ethanol solution (50%, 75%, 85%, and 95%, each for 5 min, and 100% three times for 10 min) before applying the critical-point drying (CPD) method, and were sputter-coated with gold to a thickness film before observation.

### 2.9. MSCs Condensation Examination in 2D and 3D Culture

For condensation examination in 2D, the alcian blue staining method was used. Briefly, adherent cells were fixed with 4% para-formaldehyde for 30 min, mounted with 3% acetic acid at room temperature for 10 min, and washed with PBS. Then cells were stained with alcian blue solution for 30 min to react with the sulfate groups of GAGs. For 3D examination, the live/dead staining method was used. After a seven day incubation, constructs were stained with 4 μM calcein AM (Life Technologies, Grand Island, NY, USA) and 4 μM of propidium iodide (PI, Life Technologies, Grand Island, NY, USA) for 30 min. Live cells were presented in green fluorescence by calcein AM (ex/em ~495 nm/~515 nm), and dead cells were shown in red by PI (ex/em ~540 nm/~615 nm). The cell survival rate was observed by a confocal microscope (LSM 780, Zeiss, Oberkochen, Germany).

### 2.10. Quantitative Real-Time PCR (Q-PCR)

At different time periods, total RNA was extracted from the constructs using a Total RNA Miniprep Purification Kit (GeneMark, Taichung, Taiwan). The total RNA was reverse-transcribed into complementary DNA (cDNA) by using a First Strand cDNA Synthesis kit (Thermo Scientific, Waltham, MA USA) according to the manufacturer’s protocol. The housekeeping gene was *β-actin* (NM_031144), and the primer sequences are presented in Table 1. Real-time PCR reactions were performed and monitored using the OmicsGreen qPCR Master Mix (Omics, New Taipei, Taiwan) and the ABI PRISM 7500 Sequence Detection System (Life Technologies, Grand Island, NY, USA). Briefly, 5 μL of 5× OmicsGreen qPCR Master Mix, 10 μL of primers (including forward and reverse primers), and 10 μL of cDNA templates were mixed in a final volume of 25 μL for a single reaction. The PCR primers were listed in Table 1. The relative quantitation value data of gene expression was calculated using the expression of 2^−ΔΔCt^. For all the quantitative real time PCR (Q-PCR) experiments, the values are expressed as relative fold difference in comparison to expression by monolayer cells cultured for one day after seeding.

### 2.11. Hematoxylin/Eosin and Immunohistochemical (IHC) Staining

At the end of the cultivation (days seven, 14, and 21), the constructs were removed at each time-point for histological examination. Hematoxylin and eosin staining were carried out to investigate the morphology of the tri-copolymer scaffold/rMSCs constructs, and immunohistochemical observation was made for the expression of type II collagen and aggrecan. Briefly, paraffin-embedded tissue blocks were cut into 5 μm thickness for staining. After deparaffinization and rehydration processes, endogenous peroxidases were blocked by 0.1% hydrogen peroxide (Sigma-Aldrich, St. Louis, MO, USA) in PBS solution for 10 min. For the retrieval process, nonspecific background staining was blocked by 20 μg/mL proteinase K (Sigma-Aldrich, St. Louis, MO, USA) solution and incubated 20 min at 37 °C in a humidified chamber. Primary antibodies, rabbit anti-type II collagen (Abcam, Cambridge, MA, USA) and rabbit anti-aggrecan (GeneTex, Hsinchu, Taiwan), were added with appropriate dilution on the tissue sections and incubated at 4 °C overnight. After incubation, rinse tissue sections and then incubate with SuperPicture™ Polymer Detection Kit (Life Technologies, Grand Island, NY, USA) for 10 min at room temperature. Finally, the tissue sections were revealed by 3, 3′-diaminobenzidine (DAB, Sigma-Aldrich, St. Louis, MO, USA) substrate solution. For all the tissue section staining protocols, hematoxylin was used as a counterstain on the slides.

### 2.12. Statistics Analysis

All experiments were conducted at least in triplicate, and all the data was presented as the mean±standard deviation (SD). Statistical analysis was performed for all the quantitative results using one-way ANOVA for comparing means from two independent sample groups, and *p*-values less than 0.05 were considered statistically significant.

## 3. Results

### 3.1. Characteristics of Tri-Copolymer Scaffold

**Figure 1:**
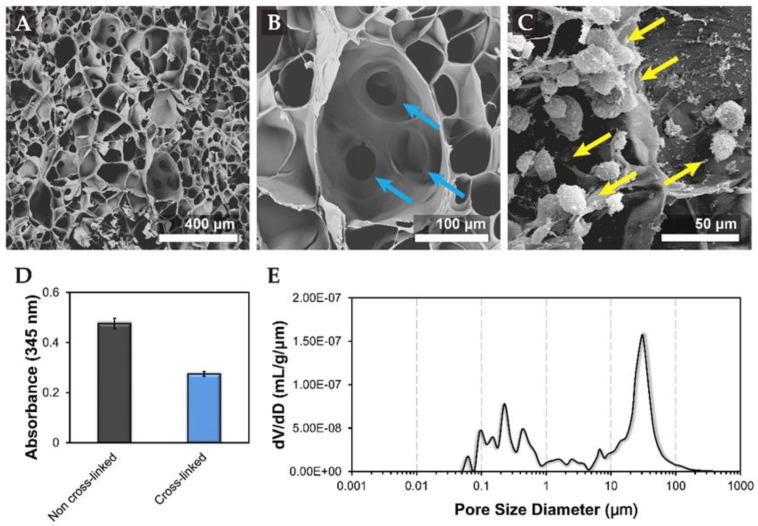
Characterization of tri-copolymer scaffolds. Scanning electron microscopic examination of the tri-copolymer scaffold showing (**A**) homogeneous pore distribution; (**B**) the blue arrow indicated interconnected pores for medium transportation; (**C**) the yellow arrow recealed that cells distributed homogeneously inside the scaffolds; (**D**) the TNBS assay revealed that the estimated degree of cross-linking was 42.2%; and (**E**) the average pore size of tri-copolymer scaffolds and interconnected porous structure inclusive were around 20 to 30 μm.

Tri-copolymer scaffold was imaged by SEM after cross-linking and lyophilization. SEM image showed the synthesized scaffold had a mean pore size of 81.85 ± 13.8 µm (Figure 1A), which would further increase after rehydration in medium and highly interconnected porous structure (Figure 1B), which allowed better oxygen and nutrient distribution through the scaffold. Supplementary Figure S1A contains an illustration of the cell seeding method, and SEM imaging performed thereafter. In brief, the cells were seeded at the center of the tri-copolymer scaffolds by pipette at a density of 5 × 10^5^ viable cells/scaffold. The SEM data demonstrated the pore size was uneven, it had large porous structures (some over 150 μm) and small interconnected pores (average of 20 to 30 μm), and cells were randomly scattered across the tri-copolymer scaffold through the interconnected pores (Supplementary Figure S1B). The porous structure not only provides passage for oxygen and nutrient transportation but also provides space as niches for cells (Figure 1C). The SEM data revealed that cells were random and scattered distributed across the tri-copolymer scaffold via the interconnected pores (Supplementary Figure S1C).

The degree of cross-linking of the tri-copolymer was estimated by the trinitrobenzene sulfonate (TNBS) assay. The optical density (OD) value of the cross-linked experimental group was 0.275 ± 0.009, while the OD value of the non-cross-linked group was 0.476 ± 0.021. Based on the OD, the degree of cross-linking was found to be 42.2% (Figure 1D). The cross-linking of pure gelatin and EDC cross-linked tri-copolymer scaffold was further assessed by Fourier transform infrared (FT-IR) spectroscopy (Supplementary Figure S2). The representative transmittance peaks, generated after carboxyl and amine group cross-linking, amide I, II, III bands (1650, 1530, and 1450 cm^−1^), ester band (1100 cm^−1^) which was generated after hydroxyl and carboxyl group were cross-linked, and the characteristic chondroitin OSO_3_ group (1060 cm^−1^) was observed.

The synthesized tri-copolymer scaffold had a porosity of 91.4% as determined by the mercury porosimetry method. The average pore size of tri-copolymer scaffolds and the interconnected porous structure was around 20 to 30 μm (Figure 1E). The highly porous structure could provide an environment for cell growth and differentiation.

### 3.2. Isolation and Characterization of Rat MSCs

**Figure 2:**
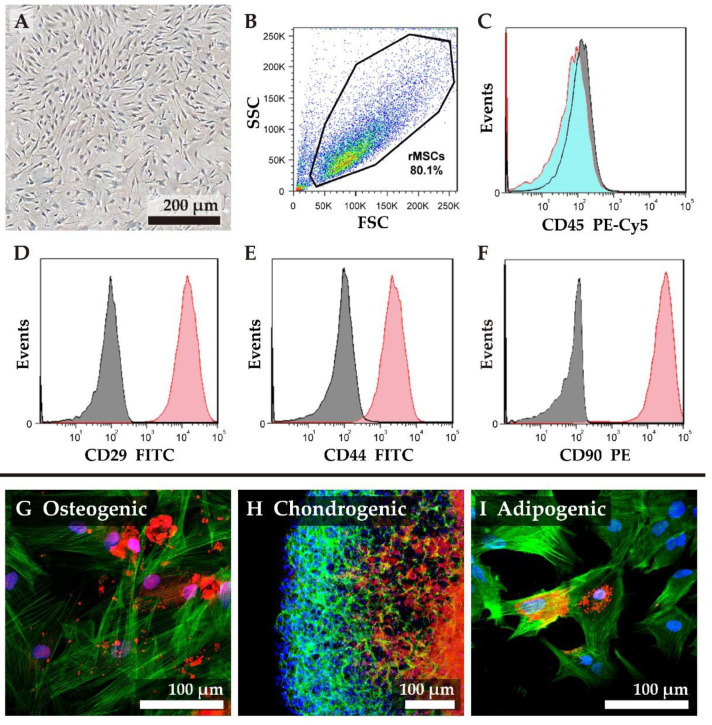
Characterization and differential abilities of rat mesenchymal stem cells (MSCs). (**A**) the image showed the morphology of rat MSCs (rMSCs) and displayed spindle-shaped; (**B**) Gate for live cells of rMSCs at passage three at FSC/SSC plot; (**C**) there was no expression of the hematopoietic markers CD45; Expression of the MSC markers (**D**) CD29, (**E**) CD44, and (**F**) CD90. Isotype controls were also included in each experiment to ensure the results (gray histogram). (**G**–**I**) showed the differential capability: (**G**) rMSCs differentiated into osteo-like cells in 14 days, and the biological apatite stained in red; (**H**) rMSCs under pellet culture treatment differentiated into chondro-like cells in 21 days, and the glycosaminoglccan stained in red; (**I**) rMSCs differentiated into adipo-like cells in 14 days, and the lipid droplets stained in red. The F-actin molecules were stained in green, and the nucleus was stained in blue.

Rat mesenchymal stem cells (rMSCs) were isolated from eight-week-old Wistar rats and propagated in vitro. The morphology of undifferentiated rMSCs was spindle-shaped (Figure 2A), and we chose the cells between passage two and four for this study. The expression of specific cell surface markers CD29, CD44, CD90, and the hematopoietic marker CD45 were analyzed by flow cytometry (Figure 2B–F). Fluorescent cell screening of undifferentiated rMSCs (Figure 2B) demonstrated that the cells were negative for CD45 expression (Figure 2C); while over 98% of cells were positive for CD29 expression (Figure 2D) and CD90 (Figure 2F). Since CD44 is a receptor for hyaluronan, chondroitin sulfate, and proteoglycans [7] that facilitates homing of rMSCs to tri-copolymer scaffolds, expression of CD44 on rMSCs was assessed. rMSCs used in this study were positive for CD44 expression (over 90%, Figure 2E).

We next studied the differentiation of rMSCs under different conditions (Figure 2G–I). In Figure 2G, rMSCs were differentiated into osteocyte like cells for seven days. Differentiation was confirmed by staining for biomineralized tissue by xylenol orange (Figure 2G). The rMSCs could also be differentiated into chondrocyte-like cells in 14 days by pellet culture, wherein the differentiation was confirmed by staining GAGs by safranin O (Figure 2H), and into adipocyte-like cells in seven days, wherein the differentiation was confirmed by staining the lipid droplets by Nile Red dye (Figure 2I). The filamentous actin (F-actin) molecules were stained in green, and the nucleus was stained in blue.

### 3.3. Determining the Optimum Dose of KGN for Chondrogenesis

**Figure 3:**
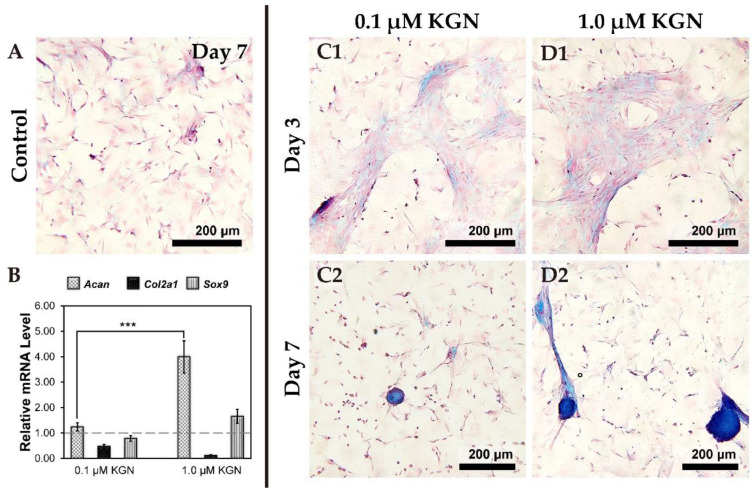
Chondrogenic differentiation and cell condensation examination in 2D condition. Alcian blue was stained for check cell condensation process; (**A**) the control group remained sparsely scattered after seven-days of cultivation. (**B**) In 2D culture, the real-time quantitative PCR (Q-PCR) results represented transcript levels related to chondrogenic gene expressions under 0.1 μM and 1.0 μM kartogen (KGN) supplement after seven days. The housekeeping gene was *β-actin*, and the monolayer cells were cultured for one day as a control group. The up-regulation of genes was normalized to rMSCs cultured for one day. When 1.0 µM KGN was added to MSCs culture for seven days, *Acan* gene expression was significantly up-regulated (*** means *p* < 0.001); *Sox9* gene expression was also up-regulated at 1.0 µM KGN group. (**C**) 0.1 μM KGN promoted condensation on day three (**C1**), and formed pellet on day seven (**C2**); and also (**D**) 1.0 μM KGN promoted condensation on day three (**D1**), and formed pellet on day seven (**D2**).

Condensation is one of the crucial processes during chondrogenesis. In order to determine the optimum concentration of KGN sufficient for chondrogenesis, we monitored the morphology, marker gene expression, and secretion of proteoglycans at different concentrations of KGN. During this experiment, rMSCs maintained spindle-shaped morphology, and remained fibroblastic throughout passages two and four used in this study. The cells remained scattered sparsely after seven days culturing of the control group (Figure 3A).

We tested two concentrations −0.1 µM and 1.0 µM KGN in 2D culture conditions for seven days and analyzed the expression of marker genes for chondrogenesis by qPCR (Figure 3B). When compared to the control group, the expression of the *Acan* gene was significantly up-regulated in both the KGN treatment groups (*p* < 0.0001). However, *Col2a1* gene expression in KGN treated groups was down-regulated when compared with the control group (monolayer culture by DMEM with 10% FBS). In addition, *Sox9* expression was slightly up-regulated in the 1.0 µM KGN group.

Staining of secreted proteoglycans by Alcian blue dye revealed that the condensation phenomenon had taken place after three days of culturing in the presence of 0.1 or 1.0 µM of KGN (Figure 3(C1,D1)), but there was no significant difference between the groups. After seven days cultivation, cell pellets could be found with Alcian blue staining both in 0.1 or 1.0 µM groups (Figure 3(C2,D2)); nevertheless, the cell pellets of the 1.0 µM group were larger in size (the size distribution was calculated by MetaMorph software). Hence, 1.0 µM KGN was used in subsequent experiments.

### 3.4. Live/Dead Staining in Static 2D and 3D Culture

**Figure 4:**
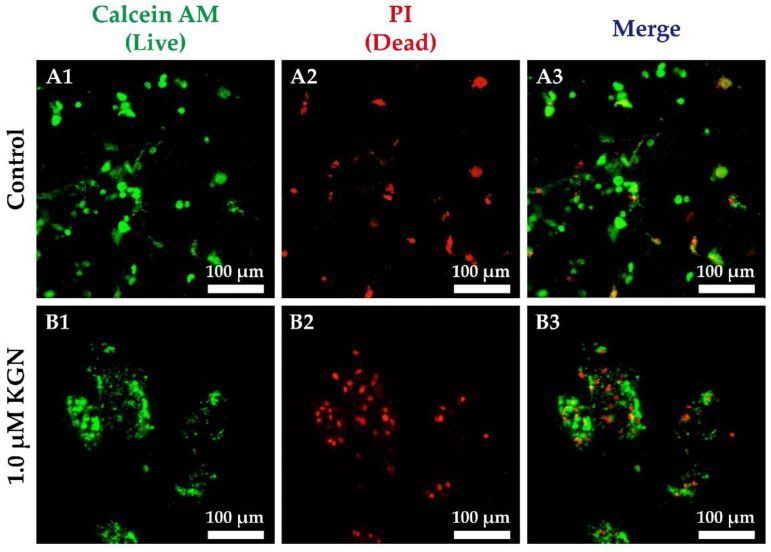
Cell condensation examination in 3D condition. The cell viability of the static condition was tested by staining with two-color fluorescent dyes, live in green and dead in red. (**A**) As shown, rMSCs sparsely scattered within the scaffold in the control group at day 14; (**B**) 1.0 μM KGN promoted condensation of rMSCs within the scaffold at day 14, and cells self-aggregated into clusters. The relative mRNA level was determined by Q-PCR, and the data were normalized by the control group and shown as mean ± standard deviation. The housekeeping gene was *β-actin*, and the monolayer cells cultured for one day after seeding acted as the control group. (**A1**,**B1**) in green represented the live cells with calcein AM dye; (**A2**,**B2**) in red (PI) indicated dead cells; (**A3**,**B3**) were the merge images.

We monitored the condensation process during chondrogenesis in static 3D culture by live/dead staining. Dead cells were found in nodule by propidium iodide (PI) staining. The rMSCs in the control group were sparsely scattered (Figure 4A), while rMSCs in tri-copolymer scaffolds condensed into nodules of around 100 µm after 1.0 µM KGN supplementation for 14 days (Figure 4B). Treatment with 1.0 μM KGN promoted condensation of rMSCs within the scaffold without significant effect on the cellular viability (Figure 4B). To evaluate the efficacy of KGN, cell proliferation in 2D culture conditions was determined by a WST-1 assay (Supplementary Figure S3).

### 3.5. Cartilage-Related Gene Expression Under 3D Static and Dynamic Culture Conditions

**Figure 5:**
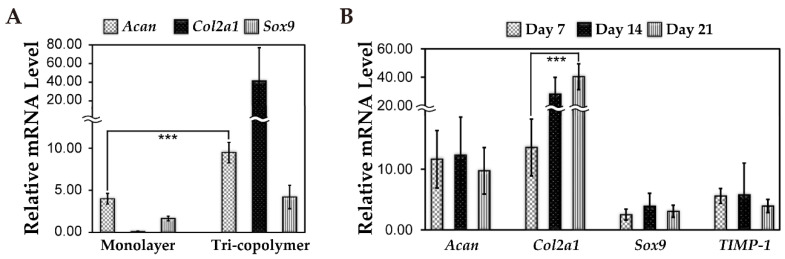
Chondrogenic differentiation in 3D condition. (**A**) When chondrogenesis markers were examined in tri-copolymer (static 3D culture), 1.0 µM KGN treatment greatly up-regulated *Acan*, *Col2a1,* and *Sox9* gene expression when compared with monolayer cells (2D culture) on day seven. (**B**) For 3D dynamic perfusion, when 1.0 µM KGN was added to rMSCs and cultured for 14 days, *Acan* gene expression was significantly up-regulated (*** means *p* < 0.001); *Sox9* gene expression was up-regulated during the first 14 days’ culture, while down-regulated in the last week’s period. However, *Sox9* expression was still more obvious when compared with that of 0.1 µM KGN treatment.

After seven days of static culture on a 3D scaffold with KGN treatment, expression of chondrogenesis-related genes was examined and compared with those in 2D culture with the presence of KGN. rMSCs cultured in the 3D scaffold up-regulated *Acan*, *Col2a1* and *Sox9* genes expression were compared to cells in 2D culture conditions (Figure 5A). This indicated that the 3D tri-copolymer scaffold could enhance the chondrogenic effects of KGN treatment. Further, *Col2a1* expression at day 21 was found to be significantly higher than at day seven in the bioreactor system (Figure 5B). This suggests that the rMSCs retained the chondrogenic phenotype up to 21 days of culturing. Expression of *TIMP-1*, a metallopeptidase inhibitor, was also up-regulated.

For evaluating the difference between the groups with or without KGN, Supplementary Figure S4 represented the live/dead staining evaluation at different time periods. The rMSCs of the control group attached on the surface of tri-copolymer scaffolds and were randomly scattered (Supplementary Figure S4A–C); on the contrary, rMSCs condensed into nodules or spheres after treating with 1.0 μM KGN supplement (Supplementary Figure S4D to S4E). As noted above, rMSCs cultured in 3D tri-copolymer scaffolds had significantly higher chondrogenic gene expression (including *Acan*, *Col2a1*, and *Sox9*) compared to the monolayer control.

### 3.6. The Comparison of KGN and TGF-β1

**Figure 6:**
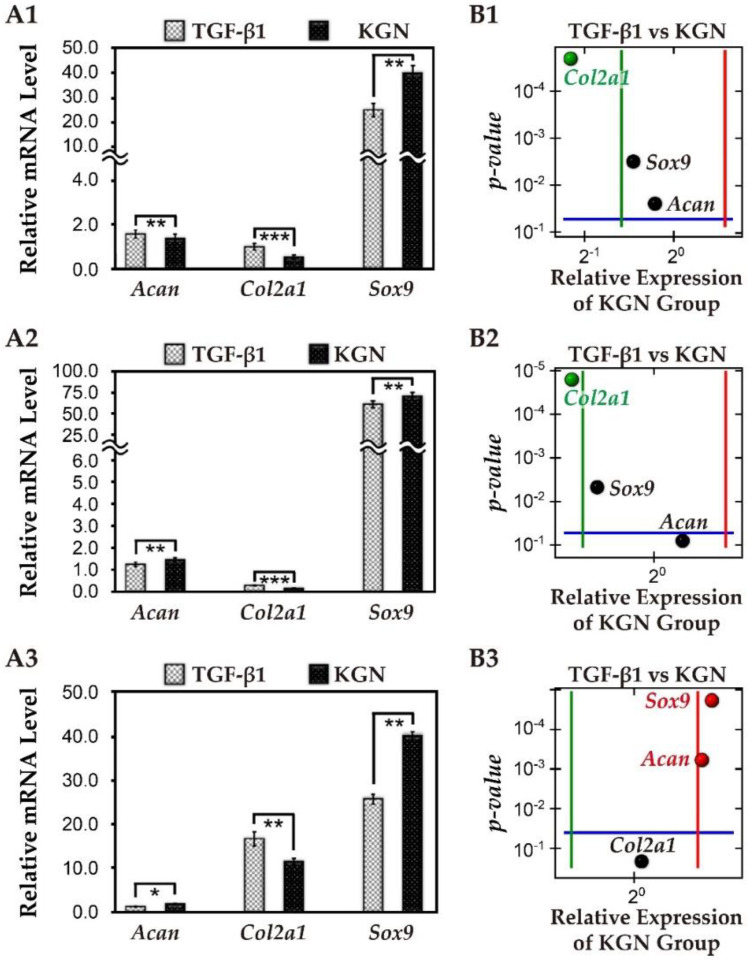
Relative chondrogenic mRNA examination in 2D condition between TGF-β1 and KGN groups. (**A**) In 2D culture, the Q-PCR results represented transcript levels related to chondrogenic gene expressions under 10 ng/mL TGF-β1 and 1.0 μM KGN supplement at day one, day three, and day seven. The relative mRNA level was determined by Q-PCR, and the data were normalized by the control group and shown as mean±standard deviation. The housekeeping gene was *β-actin*, and the monolayer cells were cultured for one day after seeding acted as the control group. (**A1**) On day one, *Acan* and *Col2a1* gene expression in the KGN group were significantly lower than TGF-β1 group; *Sox9* gene expression in the KGN group was up-regulated; (**A2**) at day three, *Col2a1* gene expression in the KGN group was significantly lower than TGF-β1 group; *Acan* and *Sox9* gene expression in KGN group were up-regulated; (**A3**) at day seven, *Col2a1* gene expression had no difference between the KGN and TGF-β1 group; *Acan* and *Sox9* gene expression in the KGN group were up-regulated (* means *p* < 0.05; ** means *p* < 0.01; *** means *p* < 0.001). (**B**) A volcano plot of relative mRNA level at day seven: the x-axis is the average fold-change ratio of the relative mRNA expression of each gene between TGF-β1 and KGN groups; the y-axis represents the *p*-value for each gene which was analyzed between the TGF-β1 and KGN groups. (**B1**) On day one, *Col2a1* in the KGN group was slightly lower than TGF-β1 group; (**B2**) at day three, *Col2a1* in the KGN group still expressed at a lower level than the TGF-β1 group; (**B3**) however, at Day seven, there was no significant difference in *Col2a1* gene, and *Acan* expression and *Sox 9* was expressed at a higher level in the KGN group (the black spot means no significant difference; the red spot means positive significant difference; the green spot means positive significant difference).

In order to compare the efficacy of KGN with that of TGF-β1, we analyzed the relative expression of chondrogenic mRNA in rMSCs cultured either in the presence of 10 ng/mL TGF-β1 or 1.0 μM KGN in 2D conditions (Figure 6). After 21-days culture, *Col2a1* gene expression of the TGF-β1 group was slightly higher than the KGN group; however, *Acan* and *Sox9* gene expression in the KGN group was significantly up-regulated.

We also examined the expression of cartilage hypertrophy-related genes such as *type I collagen* (*Col1a1*), *type X collagen* (*Col10a1*), and *Runx2* in cells treated with either KGN or TGF-β1 (Figure S4A,B). In both treatment groups, the expression of *Col1a1* and *Runx2* remained unchanged until 21 days of culturing. In the KGN-treated cells, transcripts of *Col10a1* were up-regulated at day 14, and down-regulated at day 21. In the TGF-β1 group, expression of *Col10a1* was marginally higher as compared to that in the KGN group on day seven.

Results revealed that the cells slightly proliferated in TGF-β1 and KGN groups comparing with the control group at day three and day seven; however, there was no significant difference between TGF-β1 and KGN groups during seven-days of cultivation.

### 3.7. SEM Images of Scaffold/Cell Hybrids Under Dynamic Perfusion

**Figure 7:**
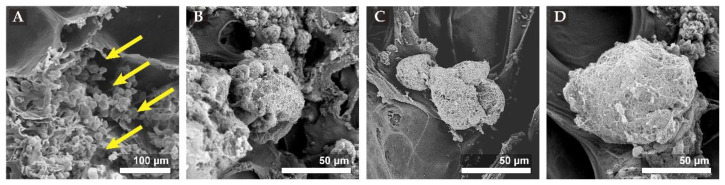
Scanning electron microscopy (SEM) images of scaffold/cells hybrid in the dynamic culture system. While KGN was added, a condensation phenomenon could be observed in the SEM images. (**A**) The image showed that the individual cells (yellow arrow site) were attached at the surface of scaffolds on day one; (**B**) scattered cells could condense and exist as clusters on day seven, at this time, the contour and morphology of individual cell were still obvious; at the (**C**) day 14 or (**D**) 21, the secreted extracellular matrix (ECM) gradually accumulated and the contour and morphology of individual cell progressively lost, but the size did not grow much as culture days expanded to 21 days (around 100 μm).

We then seeded rMSCs on the tri-copolymer scaffold and incubated them in the presence of 1.0 µM KGN for up to 21 days. The extent of condensation and ECM secretion was assessed by scanning electron microscopy. The SEM images revealed that individual cells were attached at the scaffold surface on day one (Figure 7) and underwent condensation to form clusters while still maintaining the contour and morphology of individual cells on day seven. At days 14 and 21, the secreted ECM gradually accumulated, and the contour and morphology of individual cells were progressively lost; however, the size did not increase (around 100 μm) until day 21 (Figure 7).

### 3.8. Chondrogenesis in 3D Tri-Copolymer Scaffolds and the Self-Designed Bioreactor System

**Figure 8:**
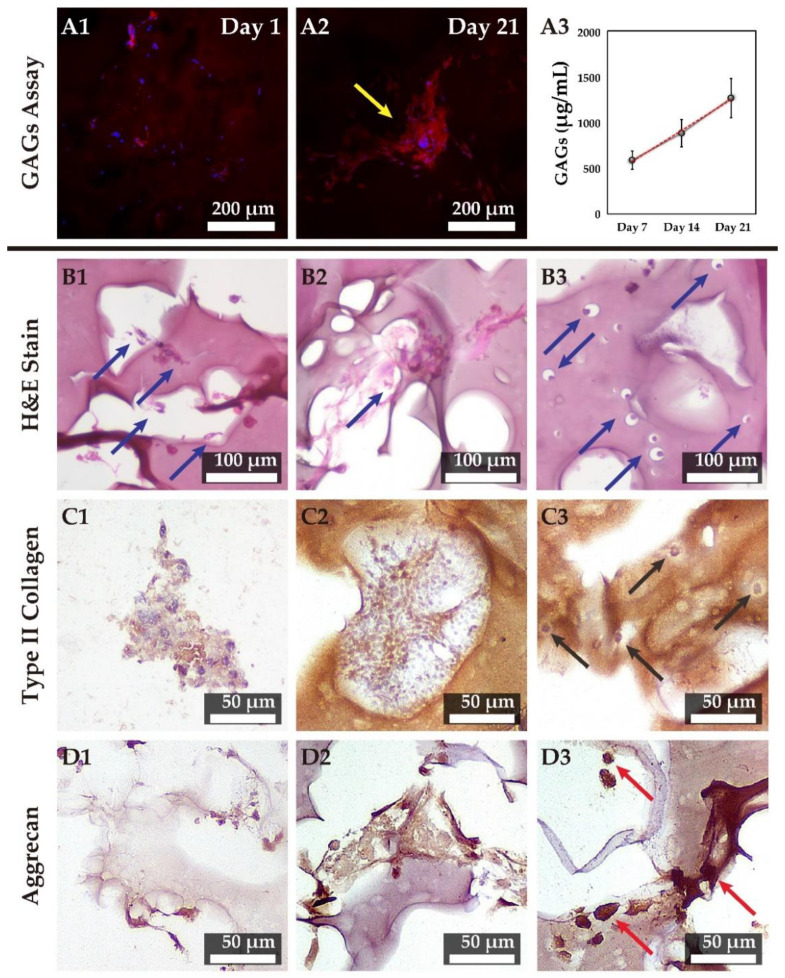
The proteoglycan examination and the immunohistochemical staining. (**A**) The confocal microscopic examination is stained by safranin O (red) and Hoechst 33342 (blue). Proteoglycans were observed on cell clusters, which correlated with safranin O results. (**A1**) The image showed that the individual cells (nucleus in blue) were inside the scaffold on day one; (**A2**) the safranin O staining of cell clusters around 100 μm could be found in the confocal image, and proteoglycan secretion within the cell cluster was clearly demonstrated after 21-days cultivation; (**A3**) and the content of glycosaminoglycans (GAGs) secreted into the cultured medium was measured by dimethylmethylene blue (DMMB) assay, we found that the GAGs contents were significantly increased with the longer culture period. (**B**) Hematoxylin and eosin staining of scaffold/cells hybrid in the self-designed bioreactor system at different time periods. Condensed cell clusters (blue arrow sites) could be found at 7 and 14 days (**B1**,**B2**); at 21 days, lacunae-like structures were embedding in the scaffold as blue arrow indicated (**B3**). The immunohistochemical observation was made for the expression of surface antigens of type II collagen and aggrecan at days one, seven and 21, and hematoxylin was used as a counterstain on the slides. (**C**) Type II collagen is accumulated in the scaffold (brown color), and the black arrow indicated the active deposition site. As mentioned above, cell clusters could be found at 7 and 14 days (**C1**,**C2**); at 21 days, lacunae-like structures were embedding in the scaffold as black arrow indicated (**C3**). (**D**) aggrecan also significantly increased as the culture period increased and accumulated inside the cell area (the red arrow site). Condensed cell clusters only were slightly aggrecan accumulation at 7 and 14 days (**D1**,**D2**); at 21 days, the accumulated aggrecan was observed as red arrow indicated (**D3**).

We next monitored secretion of proteoglycans by rMSCs cultured on tri-copolymer in the presence of KGN by staining with safranin O. In accordance with SEM data, the individual cells were scattered randomly across the scaffold on day one (Figure 8(A1)). We could clearly observe proteoglycan secretion within the cell clusters by day 21 (Figure 8(A2)). We also observed a time-dependent increase in sulfated glycosaminoglycan contents in the spent medium as measured by DMMB assay (Figure 8(A3)).

### 3.9. Hematoxylin and Eosin Staining and IHC Examination of the Tri-Copolymer Scaffold/rMSCs Constructs

In this study, we developed cartilage tissue using engineered constructs combining rMSCs, KGN, the tri-copolymer, and the self-designed bioreactor system. Supplementary Figure S6 demonstrated the effectiveness of KGN to the cell clusters. We hypothesized that KGN might induce condensation of rMSCs into cell spheres, cell nodules, or cell sheets. These cell spheres, cell nodules, or cell sheets may give rise to scaffold-cell hybrids, which then develop into lacuna-like structures within the tri-polymer. To test this, we observed cells in scaffolds cultured in the presence or absence of KGN over 21 days in culture, by hematoxylin and eosin staining (Figure S7). On day one, rMSCs were observed to be scattered throughout the scaffolds (Figure 8(B1)). Typical cartilage lacuna-like structures within cell clusters were seen in the tri-copolymer scaffolds in KGN treated cells by day seven (Figure 8(B2), blue arrow). By day 21, lacuna-like structures had attained a size of 10–20 µm (Figure 8(B3)).

For immunohistochemical staining, no area was stained positive for type II collagen at day 1 (Figure 8(C1)). At day seven, the type II collagen could be found around tri-copolymer scaffolds and within the cell clusters (Figure 8(C2)). At day 21, the lacuna-like structure showed active deposition of type II collagen (Figure 8(C3), black arrow). In contrast, aggrecan staining was mostly concentrated at the scaffold-cell hybrid at day 21 of culturing (Figure 8D). This suggests that rMSCs and tri-copolymer scaffolds may fuse together to become functional constructs for cartilage tissue engineering.

## 4. Discussion

An ideal cartilage tissue-engineered construct required three elements: a reliable and accessible cell source, the 3D scaffold that is favorable for cell attachment and chondrogenesis, and a controlled cultivation system that can transduce chondrogenic signals. Our SEM data demonstrated that the freeze-drying technique produced pores of varying sizes. Tri-copolymer scaffolds exist as non-uniform porous structures, including large pores (even over 150 μm) that provide space for cell attachment and small interconnected ones (average in 20 to 30 μm) that are essential for cell migration and nutrient transportation.

Since cartilage tissue can be engineered from a variety of cell sources, the choice of cell sources for cartilage tissue engineering is a crucial but challenging issue [35,36]. As cells derived from different sources may have varying differentiation potential, which may affect chondrogenic outcomes, at present, MSCs are one of the preferred cell sources for subsequent clinical use [37]. While using MSCs as a source, chondrogenesis is the critical stage in cartilage tissue engineering. Previously, we have demonstrated that a tri-copolymer scaffold supports the chondrocyte phenotype [4]. In this study, we hypothesized that tri-copolymer scaffolds containing hyaluronan and chondroitin-6-sulfate might provide a niche for random distribution of MSCs in 3D culture conditions, thereby supporting their chondrogenic differentiation. In this study, we provide evidence for the use of small molecules to regulate chondrogenesis in a self-designed bioreactor system.

Due to porous scaffolds facilitate better perfusion of nutrients, self-designed bioreactors with porous scaffolds provide a uniform supply of nutrients and oxygen. The closed, sealed design of the bioreactor prevents contamination of the culture, while its low cost makes it affordable for those who need such kind of cell-based treatment [29,30,31,32]. Besides, as an inexpensive heterocyclic small compound, KGN, can be easily used in the bioreactor system. Combining the use of the MSC-loaded tri-copolymer constructs with the self-designed bioreactor system, we examined the expression of chondrogenesis-related genes at different time intervals during differentiation. A previous study has indicated that long time ex vivo culture usually leads to de-differentiation of the constructs [35]. Here, we found that the hypertrophic related genes were not up-regulated even at day 21 of the differentiation protocol. Thus, the self-designed bioreactor system indeed enhances chondrogenesis of MSCs.

Chondrogenesis is the earliest stage of skeletal development and includes recruitment and migration of progenitors, condensation of progenitor cells, followed by chondrogenic cell differentiation/maturation, and cartilage/bone formation during endochondral ossification [12,38,39]. The extracellular matrix macromolecule hyaluronan can regulate coincident condensation with the onset by the expression of its own specific binding sites on the cell surface [40]. In addition, the association between hyaluronan and the cell surface binding receptors can affect cell behavior, especially cell aggregation [40,41]. The quality of cartilage tissue-engineered constructs, which are composites of cells and biomaterials, depends partially on the chemical composition of materials and on whether biomaterials can maintain the chondrocytic phenotype [42,43]. In this study, we utilized tri-copolymer scaffolds to enhance the process of chondrogenesis and formed an advanced cartilaginous construct. Hyaluronan, which is present in ECM during embryonic cartilage development, regulates cell function and helps MSC condensation. Both chondroitin and hyaluronan provide binding sites for the CD44 receptor on MSCs, to facilitate cell adhesion and regulate cell functions [9,44]. Further, scaffolds with the addition of chondroitin sulfate or hyaluronan can improve proteoglycan and type II collagen synthesis [42,45]. According to Figure 5A, KGN-induced rMSCs cultured in tri-copolymer scaffolds showed *Acan*, *Col2a1*, and *Sox9* up-regulation. And the hematoxylin and eosin staining images of MSC-based chondrogenic constructs represented lacunae-like structure formation after 21-days dynamic cultivation. The data certified that scaffolds composed of key elements of the cartilage ECM might facilitate chondrogenesis.

Recently, small molecules have emerged as candidates to regulate MSC behavior instead of growth factors. These small molecules can selectively skew the behavior of stem cells during differentiation and are relatively inexpensive, making them an attractive option for clinical use. In this study, we proposed the use of KGN, which selectively up-regulates the expression of *Acan*, *Col2a1*, *Sox9*, and *TIMP-1*, for chondrogenic differentiation [46]. Meanwhile, we noticed that KGN modulated *Sox9* and *Acan* mRNA levels were up-regulated during seven to 21-days of cultivation under 2D conditions; however, *Col2a1* gene expression was down-regulated in the KGN treated groups. Since we knew that *Col2a1* was a specific marker of late chondrogenesis, the related mRNA levels of *Col2a1* at early chondrogenesis might show a slight declining trend [47]. In contrast, it would present a sharply rising trend at chondrogenesis for maturation [13,47].

Damaged or eroded hyaline cartilage leads to progressive debilitation, affecting the quality of life across all ages in both sexes [48]. As mentioned above, mature hyaline cartilage has restricted self-repair potential due to absent innervation, insufficient vascular supply, and low mitotic capacity of chondrocytes [12,48]. Relative minor defects can be healed by chondrocyte migration, while large ones can be healed by inferior fibrocartilage formation. During chondrogenesis of MSCs, growth factors are the most important extrinsic factors in the process. Among all, transforming growth factor-beta (TGF-β) superfamily are probably the most extensively investigated [48]. However, the induction of chondrogenesis in MSCs with TGF-β leads to hypertrophic phenotype [49,50]. At this stage, chondrogenic cells exit the proliferative phase and express hypertrophic chondrocyte markers, such as *Runx2* and *Col10a1*. *Runx2* is a bone-associated transcription factor and positively regulates chondrocyte maturation towards the hypertrophic phenotype prior to ossification [38]. Besides their induction of the hypertrophic phenotype, growth factors are too expensive for large-scale use (for example, in a bioreactor system) and possess a risk of infection when applied to patients. Thus, there is a need for other inexpensive and efficacious chondrogenic signals.

To validate the chondrogenesis process in the self-designed bioreactor system, we examined the cell morphology and gene expression of the MSC-based chondrogenic constructs. We found out that, with the addition of KGN, a pellet cell mass was formed. Although the dimension of the cell mass remained constant, the cell mass showed an increasing tendency for GAG deposition. This result was further confirmed by IHC staining, while the aggrecan-positive area was concentrated on the cells themselves. In addition to the condensed cell mass, we also demonstrated the lacuna-like structure in the histological study, which is seldom formed in artificial constructs. Based on our histological observations, we found that the differentiated cells, instead of wholly synthesizing their own ECM, formed a lacuna-like structure on the scaffold materials. This result presents the clear potential of KGN usage in MSC-based cartilage tissue engineering.

**Figure 9:**
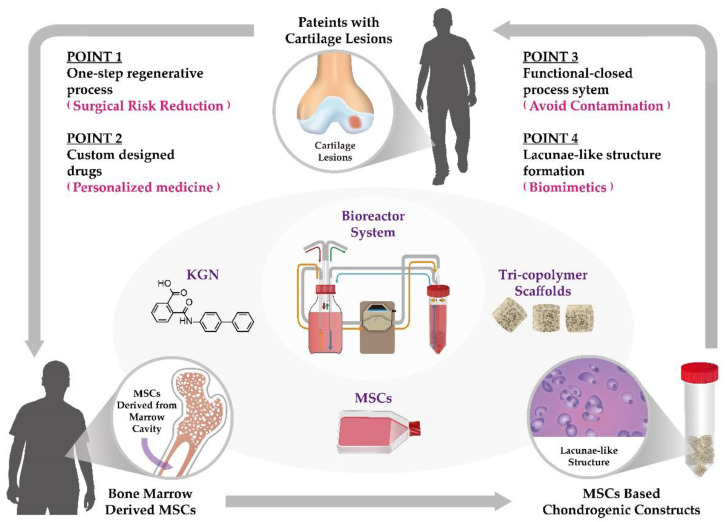
A model for MSC-based cartilage tissue engineering. In this model, it is provided with a one-step regenerative process for personalized medicine practice. For cartilage tissue engineering, MSCs are isolated from the bone marrow cavity, tri-copolymer scaffolds are performed, and KGN is added to regulate chondrogenesis. The self-designed bioreactor system can avoid contamination, and lacunae-like structures are formed and preserved inside the MSC-based chondrogenic constructs. In the future, these MSC-based chondrogenic constructs would be ready for surgical implantation. (The illustrative drawing was created by Yu-Tung Chen.).

Overall, in this study, the self-designed bioreactor system was used as a model for MSC-based cartilage tissue engineering (Figure 9). Since articular cartilage has very limited capability for self-healing, it is critical to develop new strategies for clinical management of articular cartilage lesions. For this purpose, we provide a one-step regenerative process for personalized medicine practice. In this model, MSCs were isolated from the bone marrow cavity, seeded on tri-copolymer scaffolds, and KGN was added to enhance chondrogenesis. The scaffolds are placed in the bioreactor. After 21 days of incubation, lacuna-like structures are formed in the MSC-based chondrogenic constructs. We propose that, in the future, the MSC-based chondrogenic constructs will be considered as a potential option to treat articular cartilage lesions.

## 5. Conclusions

Based on the current results, the combination of tri-copolymer/MSCs with KGN successfully induced the chondrogenic process as assessed at cellular and molecular levels. Cartilage tissue engineering may be more feasible when small molecules are used instead of growth factors to drive chondrogenic differentiation in the bioreactors. We conclude that this research presents small molecules as a viable and potent alternative to growth factors and paves the way for them to be used in combination with tissue constructs for the management of cartilage tissue injury.

## 6. Patents

Parts of the results are patents resulting from the work reported in this manuscript: US 10066208B2, TW I522468.

## Figures and Tables

**Table 1:** Primers sequences for Q-PCR.

Genes	Primers Sequences	Reference
*Acan* (NM_022190)	F-GGCCTTCCCTCTGGATTTAG	[26,33]
	R-CCGCACTACTGTCCAAC	
*Col2a1* (NM_012929)	F-CCCCTGCAGTACATGCGG	[33]
	R-CTCGACGTCATGCTGTCTCAAG	
*Sox9* (XM_003750950.1)	F-CTGAAGGGCTACGACTGGAC	[26,33]
	R-TACTGGTCTGCCAGCTTCCT	
*TIMP-1* (NM_053819)	F-TTTCCGTTCCTTAAACGGCC	[33]
	R-GATTCGACGCTGTGGGAAAT	
*β-Actin* (NM_031144)	F-GTAGCCATCCAGGCTGTGTT	[34]
	R-CCCTCATAGATGGGCAGAGT	

## Supplementary Materials

The following are available online at https://www.mdpi.com/2218-273X/11/1/115/s1, Figure S1: The cell seeding method and SEM evaluation; Figure S2: FT-IR evaluation of tri-copolymer scaffolds; Figure S3: WST-1 assay under 2D monolayer cultivation; Figure S4: Live/dead staining distinguished the difference with 3D static condition with different time periods; Figure S5: Transcript levels related to hypertrophic genes including *Col1a1*, *Col10a1*, and *Runx2* were also examined; Table S1: Primers sequences for Q-PCR; Figure S6: KGN-induced cell condensation at Day 21 with 3D dynamic perfusion; Figure S7: Typical cartilage lacunae-like structure evaluation by hematoxylin and eosin staining.
