# Supplementary material for: Kartogenin Enhances Chondrogenic Differentiation of MSCs in 3D Tri-Copolymer Scaffolds and the Self-Designed Bioreactor System
Source: Biomolecules. 2021 Jan 16;11(1):115. doi: 10.3390/biom11010115 (PMC7829855; doi:10.3390/biom11010115)
Supplement: Supplementary file 1 [file biomolecules-11-00115-s001.pdf]

biomolecules
MDPI

Supplementary Materials for

Kartogenin Enhances Chondrogenic Differentiation of MSCs in 3D Tri-copolymer Scaffolds and the Self-designed Bioreactor System

Ching-Yun Chen [1†], Chunching Li [2†], Cherng-Jyh Ke [3,4,5,6], Jui-Sheng Sun [7,8*], and Feng-Huei Lin [2,9*]

[1] Department of Biomedical Sciences and Engineering, National Central University, Taoyuan 32001, Taiwan; chingyun523@gmail.com
[2] Institute of Biomedical Engineering, College of Medicine and College of Engineering, National Taiwan University, Taipei 10002, Taiwan; R01548007@ntu.edu.tw
[3] Biomaterials Translational Research Center, China Medical University Hospital, Taichung 40202, Taiwan; fonchanwd@gmail.com
[4] Center for General Education, China Medical University, Taichung 40202, Taiwan
[5] Master Program for Digital Health Innovation, China Medical University, Taichung 40202, Taiwan
[6] Master Program in Technology Management, China Medical University, Taichung 40202, Taiwan
[7] Department of Orthopedic Surgery, National Taiwan University Hospital, Taipei 10002, Taiwan
[8] School of Medicine, College of Medicine, China Medical University, Taichung 40202, Taiwan
[9] Institute of Biomedical Engineering and Nanomedicine (I-BEN), National Health Research Institutes, Miaoli 35053, Taiwan
* Correspondence: drjssun@gmail.com (J-S S.); double@ntu.edu.tw (F-H L.)
† These authors contributed equally to this work.

The Supplementary Materials includes:

Figure S1. The cell seeding method and SEM evaluation.

Figure S2. FT-IR evaluation of tri-copolymer scaffolds.

Figure S3. WST-1 assay under 2D monolayer cultivation

Figure S4. Live/dead staining distinguished the difference with 3D static condition with different time periods.

Figure S5. Transcript levels related to hypertrophic genes including *Col1a1*, *Col10a1* and *Runx2* were also examined.

Table S1. Primers sequences for Q-PCR.

Figure S6. KGN-induced cell condensation at Day 21 with 3D dynamic perfusion.

Figure S7. Typical cartilage lacunae-like structure evaluation by H&E staining.

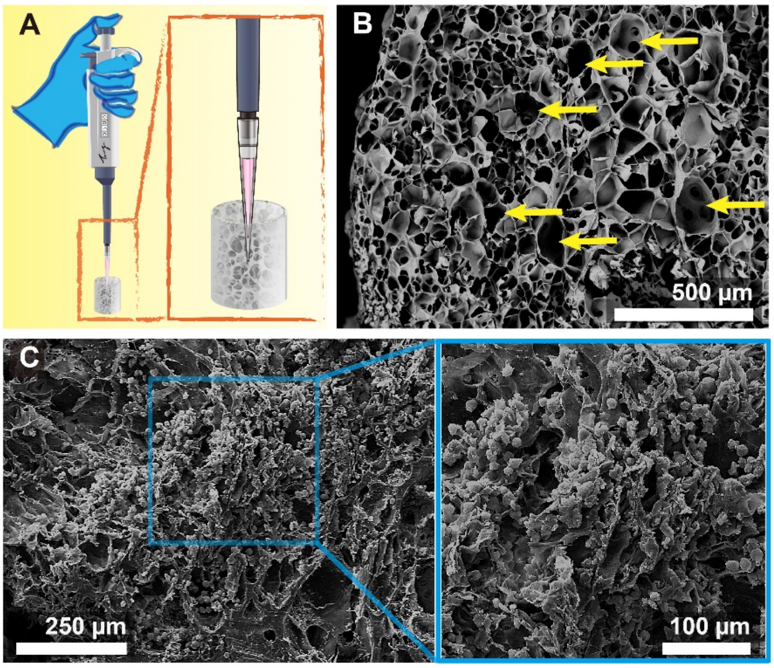

Figure S1. The cell seeding method and SEM evaluation. (A) showed the illustration of cell seeding process by holding pipet into the center of the tri-copolymer scaffolds; (B) demonstrated the pore size was performed randomly, it exists large porous structure (even over 150 µm) and small interconnected pore (average in 20 to 30 µm); (C) revealed scattered distributed across the tri-copolymer scaffold via the interconnected pores. (The illustrative drawing was created by Mr. Yu-Tung Chen.)

Materials and Methods

Scanning electron microscopy (SEM)

The protocol was described at *Section 2.8*. Briefly, the scaffold and MSC morphology inside tri-copolymer scaffolds was observed by SEM (TM 3000, Hitachi, Tokyo, Japan). Briefly, cells in scaffolds were fixed with 4% para-formaldehyde (PFA, Affymetrix, Santa Clara, CA, USA) for 2 h and 2% osmium tetroxide (OsO_4, Sigma-Aldrich, St. Louis, MO, USA A) solution for 1 h. All the samples were dehydrated in a graded ethanol solution (50%, 75%, 85%, and 95%, each for 5 min, and 100% 3 times for 10 min) before critical-point drying (CPD) method, and were sputter-coated with gold to a thickness film before observation.

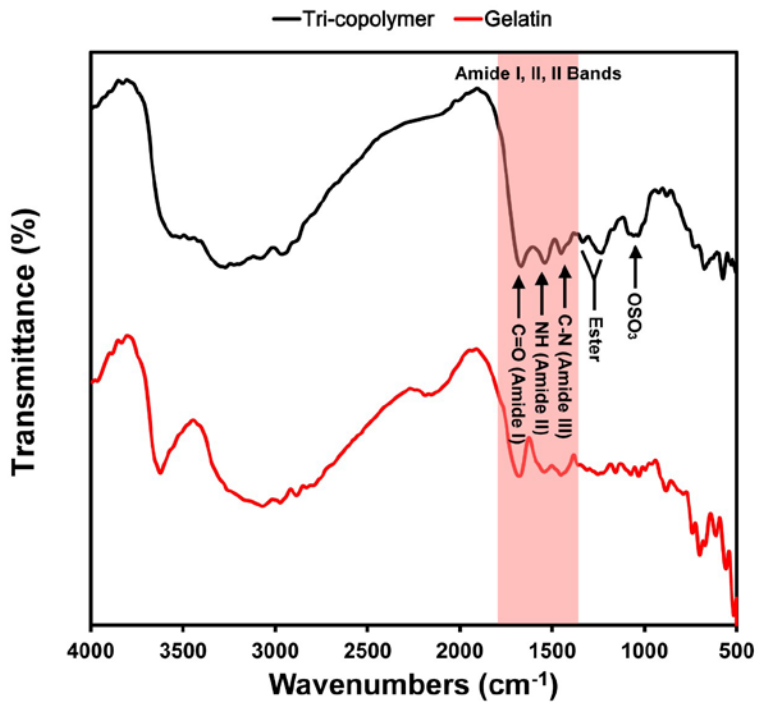

Figure S2. FT-IR evaluation of tri-copolymer scaffolds. The FT-IR data showed the transmittance peak of amide I, II, III bands (1650, 1530, and 1450 cm^{-1}), ester band (1100 cm^{-1}), OSO_3 group (1060 cm^{-1}).

Materials and Methods

Specific functional group evaluation

The specific functional group changes of tri-copolymer scaffolds were evaluated by Fourier transform infrared spectroscopy (FT-IR) as described previously [1]. Briefly, the functional groups of the tri-copolymer and pure gelatin powder were analyzed by FT-IR spectroscope (Spectrum 100 FT-IR Spectrometer, PerkinElmer, Waltham, MA, USA). The previously sample powder was added to the device of attenuated total reflection (ATR) for further analysis. The spectra and absorption bands were recorded in the wave number range of 4000–500 cm^{-1} with 16 scans per sample cycle. The absorption bands on the FT-IR spectrum were analyzed by Integrated Spectral Data Base System for Organic Compounds (SDBS).

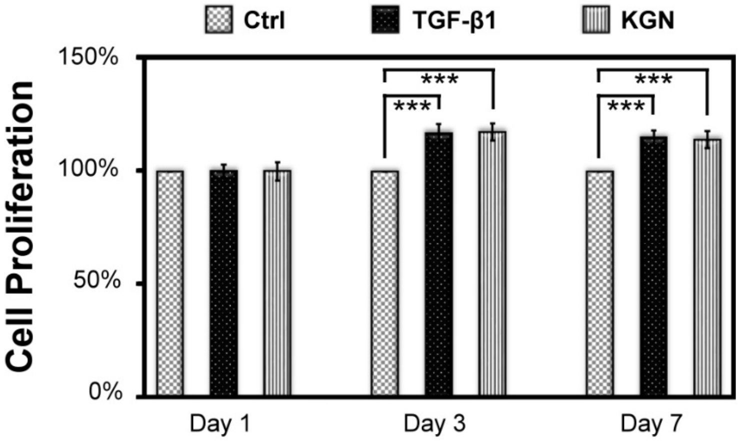

Figure S3. WST-1 assay under 2D monolayer cultivation. During 1 to 7 days' 2D cultivation, the WST-1 evaluation presented similar tendency between TGF-β1 and KGN groups. Ctrl means control group, the cells were treated with normal cell culture medium; in TGF-β1 group, the cells were treated with cell culture medium containing 10 ng/mL TGF-β1; in KGN group, the cells were treated with cell culture medium containing 1.0 μM KGN. At Day 1, there was no significant difference; at Day 3 and Day 7, the cells slightly proliferated in TGF-β1 and KGN groups (*** means $p < 0.001$). The relative proliferative level was determined by WST-1 assay, and the data was normalized by control group and shown as mean ± standard deviation.

Materials and Methods

Cell proliferation colorimetric assay (WST-1 assay)

The cell proliferation rate of 2D monolayer cells were evaluated via WST-1 assay. In brief, rMSCs were suspended in LG-DMEM with 10% FBS and then seeded into 96-well culture plate at a density of 2.5×10^3 cells/cm^2. WST-1 working solution was comprised of 10x dilution from original stock reagent with cultured medium and incubated for 1 h in the dark. The relative formazan absorbance of WST-1 reduction was measured with a multi-functional microplate reader (EnSpire™, PerkinElmer, Waltham, MA, USA) at 450 nm (690 nm was used as reference wavelength and subtracted).

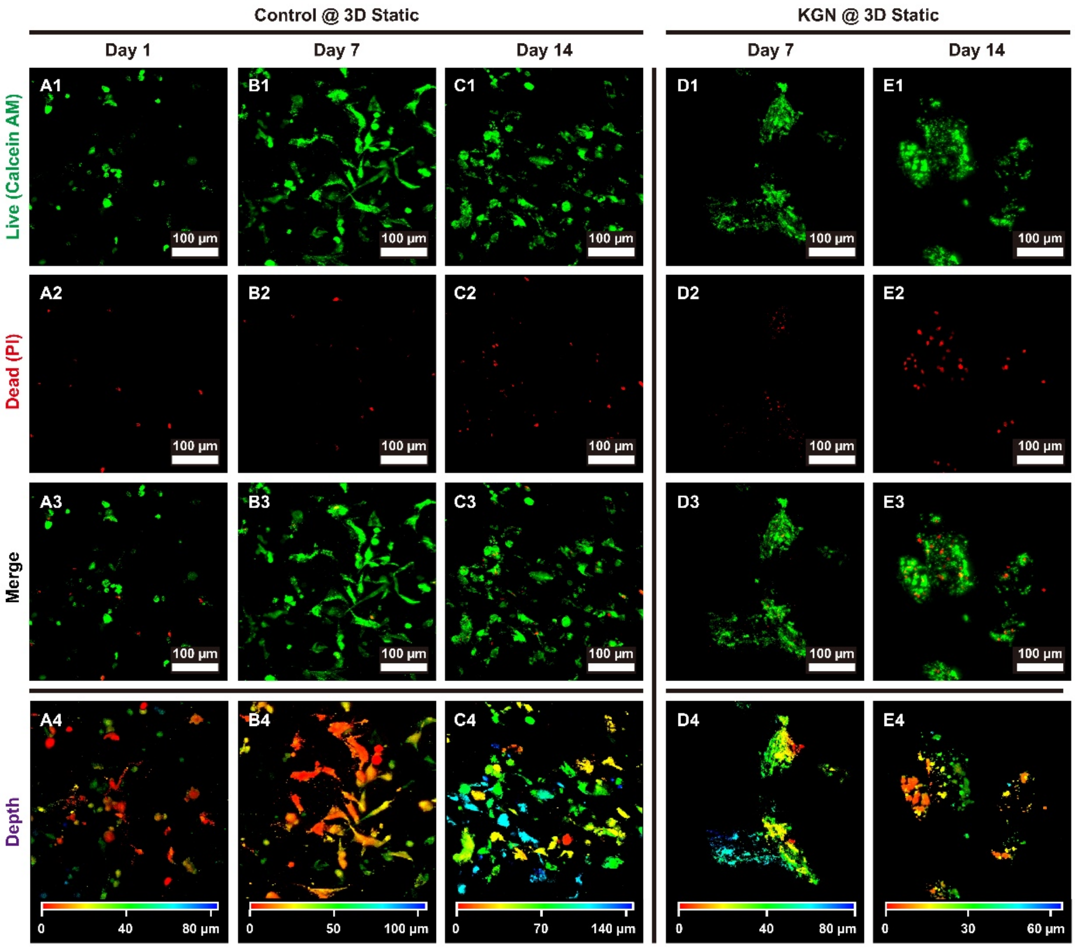

Figure S4. Live/dead staining distinguished the difference with 3D static condition with different time periods. (A1, B1, C1, D1, and E1) in green represented the live cells with calcein AM dye; (A2, B2, C2, D2, and E2) in red (PI) indicated dead cells; (A3, B3, C3, D3, and E3) were the merge images; (A4, B4, C4, D4, and E5) displayed the depth code.

Materials and Methods

Live/dead staining for 3D cell constructs under static condition

The protocol was described at *Section 2.9*. Briefly, after incubation for different time periods, constructs were stained with 4 μM calcein AM (Life Technologies, Grand Island, NY, USA) and 4 μM of PI (Life Technologies, Grand Island, NY, USA) for 30 min. Live cells were presented in green fluorescence by calcein AM (ex/em ~495 nm/~515 nm), and dead cells showed in red by PI (ex/em ~540 nm/~615 nm). The cell survival rate was observed by a confocal microscope (LSM 780, Zeiss, Oberkochen, Germany).

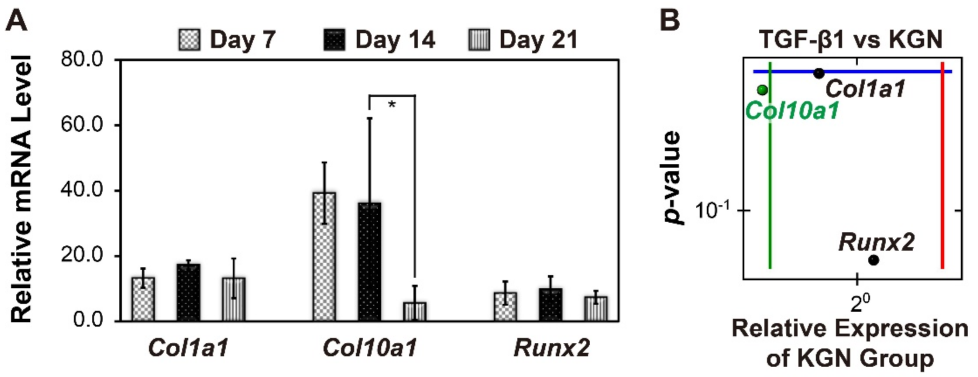

Figure S5. Transcript levels related to hypertrophic genes including *Col1a1*, *Col10a1* and *Runx2* were also examined. (A) During 7 to 21 days' 3D dynamic cultivation, the expression of *Col1a1* and *Runx2* were retained stationary in KGN group. For *Col10a1*, however, its gene expression was down-regulated at day 21 (* means $p< 0.05$). The house keeping gene was β-actin, and the monolayer cells cultured for 1 day after seeding acted as control group. The relative mRNA level was determined by Q-PCR, and the data was normalized by control group and shown as mean ± standard deviation. (B) The relative hypertrophic gene evaluation in 2D condition between TGF-β1 and KGN groups at Day 7 presented no significant difference in *Col1a1* and *Runx2* genes; however, *Col10a1* gene in TGF-β1 group presented lower level than KGN group.

Materials and Methods

Quantitative real-time PCR (Q-PCR)

The protocol was described at *Section 2.10*. Briefly, at different time period, total RNA was extracted from the constructs using Total RNA Miniprep Purification Kit (GeneMark, Taichung, Taiwan). The total RNA was reverse-transcribed into complementary DNA (cDNA) by using First Strand cDNA Synthesis kit (Thermo Scientific, Waltham, MA USA) according to manufacturer's protocol. The housekeeping gene is *β-actin* (NM_031144), and the primer sequences were presented at Table 1. Real-time PCR reactions were performed and monitored using the OmicsGreen qPCR Master Mix (Omics, New Taipei, Taiwan), and the ABI PRISM 7500 Sequence Detection System (Life Technologies, Grand Island, NY, USA). Briefly, 5 μL of 5x OmicsGreen qPCR Master Mix, 10 μL of primers (including forward and reverse primers), and 10 μL of cDNA templates were mixed in a final volume of 25 μL for single reaction. The PCR primers were listed in Table 1. The data of relative quantitation value of gene expression was calculated using the expression of $2^{-\Delta\Delta Ct}$. For all the Q-PCR experiments, the values are expressed as relative fold difference in comparison to expression by monolayer cells cultured for 1 day after seeding.

Table S1. Primers sequences for Q-PCR.

Genes	Primers Sequences	Reference
Col1a1 (RGD61817*)	F- TCCAGGGCTCCAACGAGA<br>R- CTGTAGGTGAATCCACTGTTGC	[2]
Col10a1 (XM_001053056.7)	F- CCCTATTGGACCACCAGGTA<br>R- TCTCTGTCCGCTCTTTGTGA	[2]
Runx2 (XM_34016)	F- GCCGGGAATGATGAGAACTA<br>R- AGATCGTTCAACCTGGCCACT	[2]
β-Actin (NM_031144)	F- GTAGCCATCCAGGCTGTGTT<br>R- CCCTCATAGATGGGCAGAGT	[3]

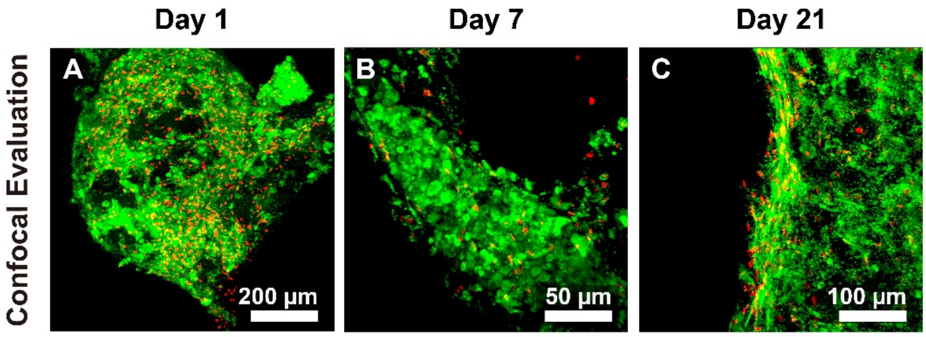

Figure S6. KGN-induced cell condensation at Day 21 with 3D dynamic perfusion. After 21 days' cultivation, KGN may induce cell condensed into (A) cell spheres; (B) cell nodule; (C) cell sheets.

Materials and Methods

Live/dead staining for 3D cell constructs under dynamic condition

The protocol was described at *Section 2.9*. Briefly, after 21-days incubation, constructs were stained with 4 μM calcein AM (Life Technologies, Grand Island, NY, USA) and 4 μM of PI (Life Technologies, Grand Island, NY, USA) for 30 min. Live cells were presented in green fluorescence by calcein AM (ex/em ~495 nm/~515 nm), and dead cells showed in red by PI (ex/em ~540 nm/~615 nm). The cell survival rate was observed by a confocal microscope (LSM 780, Zeiss, Oberkochen, Germany).

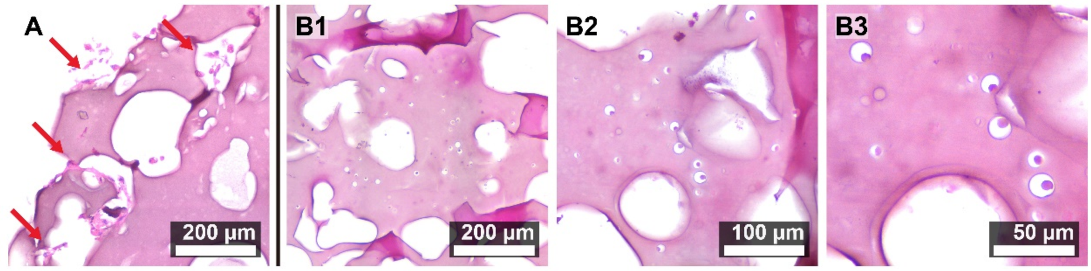

Figure S7. Typical cartilage lacunae-like structure evaluation by H&E staining. (A) Hematoxylin and eosin staining of scaffold/cells hybrid with normal culture medium in the self-designed bioreactor system at Day 21; the red arrow site presented cell distribution. (B) Hematoxylin and eosin staining of scaffold/cells hybrid in the self-designed bioreactor system at Day 21 with different power microscopic views: (B1) 100x; (B2) 200x; (B3) 400x; the lacunae-like structure distributed randomly across tri-copolymer scaffolds.

Materials and Methods

Hematoxylin/eosin (H&E) staining

The protocol was described at *Section 2.11*. In brief, at the end of the cultivation (day 7, 14 and 21), the constructs were removed at each time-point for histological examination. Hematoxylin and eosin staining were carried out for investigating the morphology of the tri-copolymer scaffold/rMSCs constructs.

Reference

1. Chen, C.-Y.; Tseng, K.-Y.; Lai, Y.-L.; Chen, Y.-S.; Lin, F.-H.; Lin, S. Overexpression of insulin-like growth factor 1 enhanced the osteogenic capability of aging bone marrow mesenchymal stem cells. *Theranostics* **2017**, *7*, 1598.
2. Grassel, S.; Ahmed, N.; Gottl, C.; Grifka, J. Gene and protein expression profile of naive and osteo-chondrogenically differentiated rat bone marrow-derived mesenchymal progenitor cells. *International journal of molecular medicine* **2009**, *23*, 745.
3. Matsuda, A.; Wang, Z.; Takahashi, S.; Tokuda, T.; Miura, N.; Hasegawa, J. Upregulation of mRNA of retinoid binding protein and fatty acid binding protein by cholesterol enriched-diet and effect of ginger on lipid metabolism. *Life sciences* **2009**, *84*, 903-907.
